# WHO antenatal care policy and prevention of malaria in pregnancy in sub-Saharan Africa

**DOI:** 10.1186/s12936-024-05037-3

**Published:** 2024-07-23

**Authors:** Bolanle Olapeju, Michael Bride, Julie R. Gutman, Katherine Wolf, Scolastica Wabwire, Deborah Atobrah, Felicia Babanawo, Otubea Owusu Akrofi, Christian Atta-Obeng, Benjamin Katienefohoua Soro, Fady Touré, Emmanuel Shekarau, Zoé M. Hendrickson

**Affiliations:** 1 Department of Preventive Medicine and Biostatistics, Uniformed Services University of the Health Sciences, Bethesda, MD USA; 2 Johns Hopkins Center for Communication Programs, Baltimore, MD USA; 3 Malaria Branch, National Center for Emerging and Zoonotic Infectious Diseases (NCEZID), Centers for Disease Control and Prevention, Atlanta, GA USA; 4 Jhpiego, Baltimore, MD USA; 5 Division of Reproductive and Maternal Health, Ministry of Health, Nairobi, Kenya; 6 Centre for Gender Studies and Advocacy, Institute of African Studies, University of Ghana, Accra, Ghana; 7 PMI/USAID IMPACT Malaria Project, Accra, Ghana; 8 National Malaria Elimination Programme, Accra, Ghana; 9 Johns Hopkins Center for Communication Programs, Abidjan, Côte d’Ivoire; 10 National Malaria Control Programme, Ministry of Health, Bamako, Mali; 11 National Malaria Elimination Programme, Abuja, Nigeria; 12 Department of Behavioral and Community Health Sciences, School of Public Health, University of Pittsburgh, Pittsburgh, PA USA

**Keywords:** Antenatal care, Malaria, Pregnancy, Sub-Saharan Africa, WHO policy

## Abstract

**Background:**

The WHO 2016 antenatal care (ANC) policy recommends at least eight antenatal contacts during pregnancy. This study assessed ANC8 uptake following policy implementation and explored the relationship between ANC attendance and intermittent preventive treatment in pregnancy (IPTp) coverage in sub-Saharan Africa following the rollout of the World Health Organization (WHO) 2016 ANC policy, specifically, to assess differences in IPTp uptake between women attending eight versus four ANC contacts.

**Methods:**

A secondary analysis of data from 20 sub-Saharan African countries with available Demographic Health and Malaria Indicator surveys from 2018 to 2023 was performed. The key variables were the number of ANC contacts and IPTp doses received during a participant's last completed pregnancy in the past two years. Pooled crude and multivariable logistic regression models were used to explore factors associated with attendance of at least four or eight ANC contacts as well as receipt of at least three doses of IPTp during pregnancy.

**Results:**

Overall, only a small proportion of women (median = 3.9%) completed eight or more ANC contacts (ANC8 +). Factors significantly associated with increased odds of ANC8 + included early ANC attendance (AOR: 4.61: 95% CI 4.30—4.95), literacy (AOR: 1.20; 95% CI 1.11—1.29), and higher wealth quintile (AOR: 3.03; 95% CI 2.67—3.44). The pooled estimate across all countries showed a very slight increase in the odds of IPTp3 + among women with eight (AOR: 1.06; 95% CI 1.00—1.12) compared to those with four contacts. In all but two countries, having eight instead of four ANC contacts did not confer significantly greater odds of receiving three or more doses of IPTp (IPTp3 +), except in Ghana (AOR: 1.67; 95% CI 1.38—2.04) and Liberia (AOR: 1.43; 95% CI 1.18—1.72).

**Conclusion:**

Eight years after the WHO ANC policy recommendation, all countries still had sub-optimal ANC8 + coverage rates. This paper is a call to action to actualize the vision of the WHO and the global malaria community of a malaria free world. Policies to improve ANC and IPTp coverage should be operationalized with clear actionable guidance and local ownership. Study findings can be used to inform multi-level policy, programmatic, and research recommendations to optimize ANC attendance and malaria in pregnancy prevention, thus improving maternal and child health outcomes, including the reduction of malaria in pregnancy.

**Supplementary Information:**

The online version contains supplementary material available at 10.1186/s12936-024-05037-3.

## Background

### Malaria in pregnancy

Malaria continues to be a global health priority, with an estimated 608,000 deaths in 2022, primarily in sub-Saharan Africa [1]. Malaria in pregnancy [MIP] carries substantial risks for mothers and unborn children, increasing maternal anemia, and adverse birth outcomes, including low birthweight, preterm birth, stillbirth, and maternal and infant mortality [2]. To prevent these adverse consequences, the World Health Organization [WHO] recommends a three-pronged approach to address MIP: the use of insecticide-treated nets [ITNs], intermittent preventive treatment in pregnancy [IPTp], and prompt and effective case management [1]. All women at risk are supposed to receive preventive interventions, including the use of ITNs and, for women in areas of moderate to high transmission, the use of IPTp [3]. However, the coverage of interventions to prevent malaria in pregnancy remains low, with less than half of all pregnancies at risk receiving the recommended three or more doses of IPTp. At the same time, the malaria burden remains high, with an estimated 12.7 million pregnancies in the WHO African Region affected in 2022 [1].

### Antenatal care

In many malaria endemic countries, ITNs and IPTp are routinely implemented through antenatal care (ANC) services. ANC remains an important intervention relevant to all maternal and child health strategic priorities as it provides a platform for primary healthcare and integration across systems of care, fosters linkages between the community and facility, and ensures referral to other networks of care as needed. ANC also offers opportunities for health promotion, screening, and diagnosis [4]. While the benefits of ANC are well known [2, 4, 5]. ANC attendance has historically been suboptimal in many malaria endemic settings in sub-Saharan Africa [6–8].

### WHO antenatal care policy

The WHO proposed the Focused Antenatal Care (FANC) programme in 2001, a model of four ANC visits throughout pregnancy: one in each trimester and two in the third trimester, with delivery of IPTp during the second, third, and fourth ANC visits [9]. The FANC model was recommended based on a large-scale cluster randomized trial that demonstrated no differences in maternal and newborn outcomes comparing FANC to the preceding traditional ANC service model, which included anywhere from seven to sixteen ANC visits.

In 2016, this policy was revised per additional research suggesting that reducing the number of ANC visits to four could carry increased risk of fetal death late in pregnancy [3, 9, 10]. As a result, the updated 2016 ANC policy focuses on improving care during the third trimester. Recommendations emphasize that ANC providers should reduce preventable morbidity and mortality rates through systematic monitoring of maternal and fetal well-being, particularly in relation to hypertensive disorders and other detectable conditions in this critical period. The policy added three more visits to the third trimester for a total of five third trimester contacts in contrast to the two contacts in the third trimester in the FANC model. Thus, the 2016 model includes a total of eight ANC contacts, with a recommendation for a maximum of six doses of IPTp to be administered in the second, third, fourth, fifth, and seventh visits (Table 1). In addition, the WHO used the 2016 update to shift from use of the term “ANC visit” to “ANC contact”. The term “contact” is meant to be adapted to local contexts to include ANC encounters with community outreach programmes and lay health worker involvement [3]. Under the 2016 recommendations, a routine ANC visit is considered a contact. As of 2020, 34 countries in the WHO African Region have adopted the WHO 2016 ANC model, including several countries in the process of implementation [11].

**Table 1:** WHO ANC models and relevant malaria in pregnancy interventions

	FANC model	2016 ANC model	Malaria in pregnancy interventions
1st trimester	Contact 1: 8–12 weeks	Contact 1: up to 12 weeks	Provide ITN Counsel on ANC attendance, malaria prevention and treatment
2nd trimester	Contact 2: 24–26 weeks	Contact 2: 20 weeks Contact 3: 26 weeks	IPTp1^a^ after 13 weeks
			IPTp2 IPTp3
3rd trimester	Contact 3: 32 weeks Contact 4: 36– 38 weeks	Contact 4: 30 weeks Contact 5: 34 weeks Contact 6: 36 weeks Contact 7: 38 weeks Contact 8: 40 weeks	IPTp4 IPTp5 – IPTp6 –

^a^WHO recommendations: IPTp is safe from the second trimester until delivery. IPTp should be given by directly observed treatment. Pregnant women should receive malaria interventions as appropriate, even when they come at weeks not designated in the contact schedule. Side effects of IPTp should be discussed openly and managed during ANC contacts

### Study rationale and objective

Given that the WHO 2016 ANC policy has been in effect for more than seven years, it is important to review current policies and programmes to determine if they have resulted in the desired levels of ANC coverage, and explore sustainability and scalability of effective policies and interventions. The study objective is to explore the relationship between ANC attendance and IPTp coverage in sub-Saharan Africa following the rollout of the WHO 2016 ANC policy; specifically, to assess differences in IPTp uptake comparing women attending eight versus four ANC contacts. Study findings can be used to inform multi-level policy, programmatic, and research recommendations to optimize ANC attendance and malaria in pregnancy prevention, thus improving maternal and child health outcomes, including the reduction of malaria in pregnancy.

## Methods

### Study design

This study analysed secondary data from Demographic Health Surveys (DHS) and Malaria Indicator Surveys (MIS) from a total of 20 countries that account for over 90% of the global malaria burden [1]. Data were downloaded with permission from the DHS Program web site, http://www.dhsprogram.com. The DHS and MIS are standard cross-sectional surveys that use a nationally representative multi-cluster sampling design to estimate several malaria related outcomes, including malaria prevalence among children under five and antenatal care attendance of pregnant women [12].

### Data source

This study focused on all countries with available DHS and MIS data dating from January 2018 to August 2023. The cutoff date of January 2018, an estimate of two years since the WHO 2016 ANC policy, was based on the fact that the surveys collect pregnancy data from women’s most recent pregnancy in the past two years preceding the survey. As a result, the data analysed here reflect the context after the official change in WHO policy in 2016. Only women who had a live birth in the last two years preceding the survey were included in the analysis. A total of 20 surveys from 20 countries from three geographic zones (Central, East and West Africa) per United Nations methodology [13] were included in the analysis as described in Additional Table 1.

### Variables

The outcomes of interest were the number of ANC contacts and IPTp doses received during a participant's last pregnancy in the past two years. Number of ANC contacts was explored as (i) a continuous variable; (ii) a categorical variable (0–3, 4–7, and 8 + contacts) and; ii(i) binary variables (≥ 1 contact versus not; ≥ 4 contacts versus not; and ≥ 8 contacts versus not). IPTp doses were explored as (i) a continuous variable and; (ii) as binary variables (≥ 1, ≥ 3 and ≥ 6 doses versus not, respectively). Covariates included in the analysis included early first ANC attendance (in the first trimester versus not), age group (15–24, 25–34, and 35 + years), residence (urban vs. rural), religion (Christian vs. other), reading literacy (no vs. yes), education level (primary or less vs. above primary), ITN use was defined as whether the participant slept under a mosquito net the night before (no vs. yes), sex of the head of household (male vs. female), water source of household (unimproved vs. improved), wealth quintile (first (lowest) to fifth (highest)), and mean number of children under 5 in the household. Some variables were not available in individual countries and are labelled as not applicable (N/A) in the tables.

### Analysis

Stata version 17 (Stata Corporation, College Station, TX, USA) was used for data management and analysis. The data were weighted using the svyset command in STATA to make the data representative of the study population for each country. Country-level chi-square tests were used to explore the associations between the various outcomes. Two-way plots were used to graph the number of ANC contacts and IPTp doses in each study country. Pooled crude and multivariable logistic regression models were used to explore factors associated with attendance of at least four or at least eight ANC contacts among all included countries. The following covariates which were available across all study countries were included in the model: early first ANC attendance (in the first trimester versus not), country, age group (15–24, 25–34, and 35 + years), residence (urban vs. rural), reading literacy (no vs. yes), sex of the head of household (male vs. female), number of children under 5 years in the household, water source (improved vs. unimproved), and wealth quintile (first (lowest) to fifth (highest)). Pooled and country-level multivariable logistic regression models were also used to explore receipt of at least three doses of IPTp during pregnancy. In the pooled model, covariates included number of ANC contacts (grouped into 4–7 as the reference, 0–3, and 8 + contacts), ANC attendance in the first trimester, age group, residence, religion, reading literacy, sex of household head, number of children under five in the household, and wealth quintile. Country-level models included all the aforementioned covariates in addition to religion and ITN use where available.

## Results

### Description of the study population

Table 2 describes the study population. Across study countries, about a third of respondents were aged 15 to 24 years (median = 34.3%, range = 22.9% (Ghana) to 45.1% (Madagascar); Table 2). Most respondents lived in rural areas (median = 64.8%, range = 8.4% (Gabon) to 85.4% (Madagascar)) and in households with improved water sources (median = 65.8%, range = 31.7% (Burkina Faso) to 89.4% (The Gambia). Fewer respondents had a primary education (median = 30.2%, range = 11% (Niger) to 78% (Gabon)). In all included countries, more than 45% of households had at least two children under 5 years of age (median = 59.5%, range = 45.6% (Ghana) to 86% (Mali)). ITN use varied by country, ranging from 23% in Mauritania to 90% in Niger with a median of 67.7%. Female-headed households were in the minority, ranging from 4% in Mali to 37% in Mauritania with a median of 20.8%.

**Table 2:** Description of the study population

Country	% 15–24 years old	% Rural resident	% Christian	% Reading literate	% ≥ Primary education	% ITN use	% ≥ 2 children under 5 years in HH	% Female HoH	% Improved HH water source	% Richest HH
Burkina Faso	33.4	74.4	30.2	29.3	18.7	77.2	68.6	8.8	31.7	17.9
Cameroon	35.6	55.8	66.3	61.2	42.5	68.5	69.6	18.9	66.0	15.1
Côte d’Ivoire	33.4	49.5	44.0	33.9	21	N/A	55.4	16.9	78.9	15.8
Gabon	32.1	8.4	82.5	86.7	78.1	31.6	59.4	32.4	81.9	17.0
The Gambia	27.5	33.5	N/A	32.7	37.4	49.2	80.6	16.0	89.4	17.8
Ghana	22.9	56.1	75.6	49.5	57.9	54.7	45.6	32.3	59.5	17.0
Guinea	39.6	72.7	98.1	20.2	16.3	N/A	72.5	13.0	72.8	16.8
Kenya	34.4	63.8	89.3	84.1	51.4	61.2	75.8	27.6	58.6	19.0
Liberia	39.9	44.8	82.4	46.4	40.7	50.3	49.0	33.5	67.6	17.6
Madagascar	45.1	84.5	62.0	69.2	35.5	69.8	50.7	17.3	40.9	14.7
Mali	34	80.2	2.3	20.1	17.2	81.5	85.6	4.3	78.8	17.2
Mauritania	28.2	58.9	N/A	48.7	18.9	23.4	75.4	36.6	72.1	14.8
Mozambique	40.8	71.1	N/A	44.7	22.9	83.3	50.9	28.7	61.2	15.7
Niger	36.3	83.6	N/A	12.5	11.0	90.2	71.5	6.8	51.3	18.7
Nigeria	27.7	72.2	34.3	46.7	39.4	48.1	69.4	6.6	63.7	18.6
Senegal	28.3	61.8	2.0	34.7	19.4	68.5	78.9	26.3	79.9	17.6
Sierra Leone	34.6	64.6	100.0	32.4	32.6	66.8	57.4	23.4	57.0	15.8
Tanzania	35.9	72.8	N/A	73.4	23.6	75.2	59.5	21.3	62.7	18.4
Uganda	31.7	69.6	85.2	60.6	27.8	73.8	57.4	25.8	75.4	17.5
Zambia	40.4	64.9	98.4	57.7	39.9	58.5	58.1	20.3	65.6	16.3
Median	34.3	64.8	75.6	46.6	30.2	67.7	59.5	20.8	65.8	17.1

*HH* households, *HoH* Head of Household, *ITN* insecticide-treated nets, *N/A* not available

### Antenatal care contacts and intermittent preventive treatment of malaria

Table 3 presents ANC and IPTp indicators across study countries. The overwhelming majority of women with a recent pregnancy had at least one ANC contact (ANC1 + ; median = 96.9%, ranging from 76.3% in Nigeria to 99.7% in The Gambia). Having four or more ANC contacts (ANC4 +) was notably lower (median 60.8%); Mauritania had the lowest proportion (39.5%), while Ghana had the highest (90.5%). In contrast, having eight or more ANC contacts (ANC8 +) was low across most countries (median = 3.9%). The highest rates were observed in Sierra Leone (21.8%), Liberia (26.9%), and Ghana (42.6%), while in Niger, Senegal, and Burkina Faso, less than one percent of women had eight ANC contacts. Early first ANC attendance (within the first trimester) varied widely across countries, from 18.5% of women in Mozambique to 70.4% in Liberia. In all countries, at least 50% of pregnant women received at least one dose of IPTp. The Gambia reached 96% of pregnant women with at least one dose of IPTp (IPTp1 +). Receipt of at least three doses of IPTp (IPTp3 +), as recommended by the WHO, ranged from 10.2% in Mauritania to 58.7% in Ghana. Receipt of six or more doses of IPTp (IPTp6 +), which is possible with the WHO 2016 model, was low, with most countries at less than one percent. Across all ANC and IPTp indicators, Ghana had the highest rates, with over 90% attending at least four ANC contacts and receiving one IPTp dose. Ghana also had the highest percentage of pregnant women attending at least eight ANC contacts and receiving at least three IPTp doses.

**Table 3:** ANC and IPTp indicators across study countries

Country	% Number of ANC contacts			% Early (1st trimester) first ANC contact	% Number of IPTp doses		
	ANC1 +	ANC4 +	ANC8 +		IPTp1 +	IPTp3 +	IPTp6 +
Burkina Faso	98.9	72.2	0.9	54.1	91.6	56.0	1.8
Cameroon	86.9	62.8	6.6	39.1	71.9	31.4	5.0
Côte d’Ivoire	95.6	56.7	3.9	39.5	78.7	32.6	4.8
Gabon	96.9	77.4	12.3	66.8	76.5	35.7	4.2
The Gambia	99.7	78.8	4.5	42.1	96.1	51.2	1.2
Ghana	97.6	90.5	42.6	66.5	90.4	58.7	1.3
Guinea	94.5	58.8	6.7	37.5	87.0	49.9	5.6
Kenya	94.0	60.3	3.4	29.5	36.8	22.5	2.6
Liberia	98.6	87.2	26.9	70.4	86.4	39.0	2.5
Madagascar	88.1	57.8	2.0	28.9	50.1	30.7	0.9
Mali	85.9	44.7	1.8	35.7	77.6	34.7	1.7
Mauritania	89.2	39.5	3.9	57.9	52.2	10.2	2.0
Mozambique	94.0	51.0	1.8	18.5	79.4	39.7	4.1
Niger	93.8	44.4	0.2	24.1	81.5	25.1	0.2
Nigeria	76.3	51.4	11.8	25.7	59.0	30.9	3.6
Senegal	98.2	53.6	0.4	59.5	91.3	18.8	0.1
Sierra Leone	98.7	79.8	21.8	44.1	89.5	34.4	4.2
Tanzania	89.7	64.7	3.0	34.3	79.2	31.1	0.7
Uganda	98.4	57.3	1.4	33.5	87.2	39.5	1.2
Zambia	98.8	63.3	1.3	37.6	93.6	58.2	1.0

**Fig. 1:**
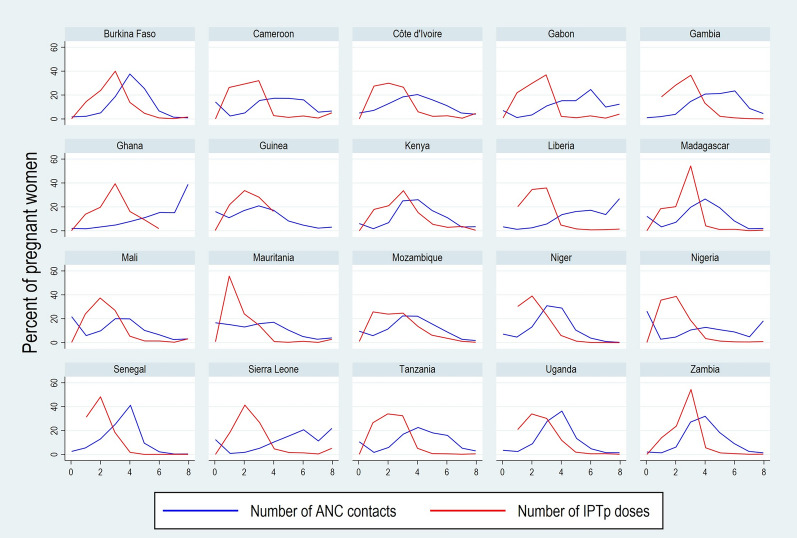
Number of ANC Contacts and IPTp Doses across Study Countries

The pattern of ANC contacts as well as IPTp doses received varied markedly across countries (Fig. 1). Ideally, a higher percentage of women should have eight contacts compared to those with zero to seven contacts, per the WHO policy. This was clearly seen in Ghana and to some extent in Liberia. However, other countries had a bell-shaped curve of ANC attendance including Burkina Faso, Côte d’Ivoire, The Gambia, Guinea, Kenya, Niger, Uganda and Zambia. Additionally, Madagascar, Mali and Mozambique and Tanzania also had somewhat bell-shaped curves but with higher rates of no ANC attendance.

In several countries, a greater proportion of women received 3 doses of IPTp than only 1 or 2 doses, this exceeded 50% in only 3 counties (Ghana, Madagascar, Zambia). In Mali and Mozambique, similar proportions of women received one, two, and three doses of IPTp, while in other countries—Côte d’Ivoire, Mauritania, Sierra Leone and Uganda—the proportion of women receiving two doses was greater than the proportion receiving three doses of IPTp.

### Factors associated with antenatal clinic attendance

Multiple factors were significantly associated with increased odds of having at least four or eight ANC contacts (Table 4). Early first ANC attendance was associated with higher odds of ANC4 + (AOR: 4.94; 95% CI 4.76—5.12) and ANC8 + (AOR: 4.61: 95% CI 4.30—4.95). Women 25 years or older had higher odds of ANC4 + and ANC8 + compared to women aged 15 to 24 years. Compared to women who were not literate, literate women were 1.39 (95% CI 1.33—1.44) and 1.20 (95% CI 1.11—1.29) times more likely to have ANC4 + and ANC8 + , respectively. There was a dose–response relationship between wealth quintile and ANC attendance with the odds of four and eight contacts increasing with each additional quintile of wealth. The odds of women in the richest quintile having at least four or eight ANC contacts were 2.42 times (95% CI 2.27—2.59) and 3.03 times (95% CI 2.67—3.44) higher, respectively, than those in the poorest wealth quintile (p < 0.001 for both). In comparison, there was a significant inverse relationship between the number of children under five in the household and ANC attendance. Women with more than one child under five years old had lower odds of having at least four (AOR: 0.89; 95%: 0.86—0.92) or eight (AOR: 0.83: 95% CI 0.78—0.89) ANC contacts than women with only one or no children. Rural versus urban residence was not significantly associated with ANC attendance overall.

**Table 4:** Pooled Multi-Country Correlates of ANC4 and ANC8

Characteristics	ANC4+		ANC8+	
	AOR^a^	95% CI	AOR^a^	95% CI
ANC in first trimester (ref=no)	4.94***	4.76 5.12	4.61***	4.30 4.95
Age in years (ref:15–24)				
25–34	1.11***	1.07 1.15	1.18***	1.09 1.27
≥35	1.05*	1.01 1.10	1.21***	1.11 1.32
Rural residence (ref: no)	1.00	0.96 1.05	0.95	0.88 1.04
Reading literate (ref: no)	1.39***	1.33 1.44	1.20***	1.11 1.29
Female head of household (ref: no)	1.04	1.00 1.08	1.06	0.98 1.14
≥2 children under five (ref: no)	0.89***	0.86 0.92	0.83***	0.78 0.89
Wealth quintile (ref: poorest)				
Poorer	1.29***	1.24 1.35	1.17**	1.06 1.30
Middle	1.58***	1.50 1.66	1.45***	1.30 1.61
Richer	1.82***	1.72 1.92	1.86***	1.66 2.09
Richest	2.42***	2.27 2.59	3.03***	2.67 3.44

AOR Adjusted Odds Ratio, CI Confidence Interval, Ref reference

^a^Adjusted for Country (estimates not shown), early first ANC, age, residence, reading literacy, sex of head of household, number of children under five in household and wealth quintile

*p<0.05; **p<0.01; ***p<0.001

### Relationship between four or eight antenatal clinic contacts and IPTp

**Fig. 2:**
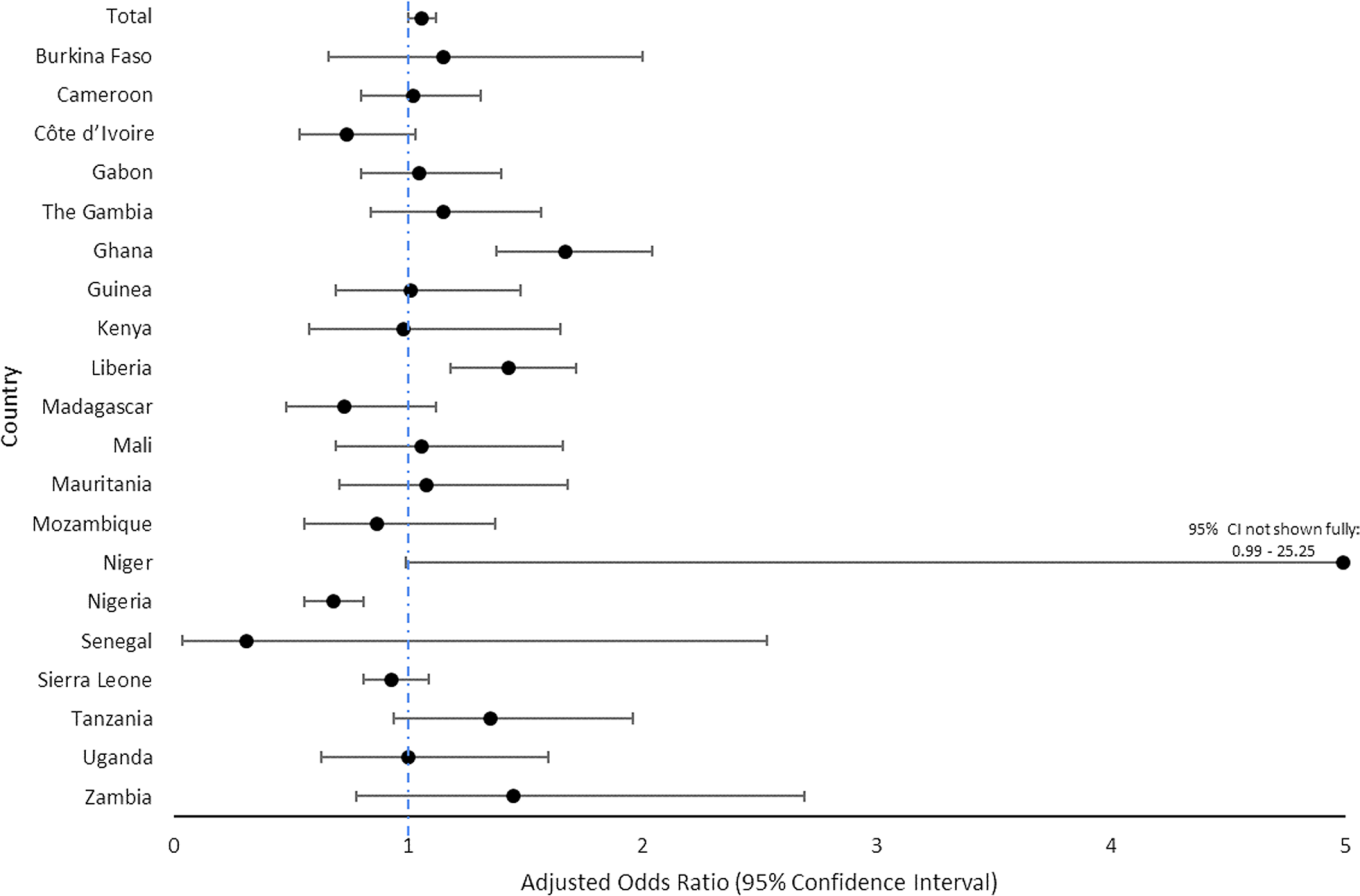
Odds of IPTp3 among Women with at Least Eight Compared to Four ANC Contacts: Overall and Country-level Estimates

While the pooled estimate across all countries revealed increased odds of receiving three or more doses of IPTp among women with eight compared to four ANC contacts (AOR: 1.06; 1.00—1.12), this was not the case in most country specific analyses (Fig. 2; Supplemental Table 2). Only in Ghana (AOR: 1.67; 95% CI 1.38—2.04) and Liberia (AOR: 1.43; 95% CI 1.18—1.72), where more than a quarter of women had ANC8 + , did eight or more contacts result in higher odds of IPTp3 + . Notably, no difference in IPTp3 + coverage was seen between women with four versus eight ANC contacts in Sierra Leone, a country with > 20% of women with eight or more ANC contacts. In addition, Nigeria had a negative correlation between ANC visits and IPTp uptake, as did several other countries although the results were not significant.

## Discussion

This study explored the coverage of eight ANC contacts as well as the relationship between ANC and IPTp in sub-Saharan Africa following the WHO 2016 ANC policy recommendation. Given that each ANC contact after the first trimester is an opportunity to deliver IPTp, it would be expected that women with more ANC contacts would be more likely to achieve at least three doses of IPTp. While the pooled estimates supported a slightly higher likelihood of receiving IPTp3 among women with eight compared to four ANC contacts, this relationship was not consistently seen at the country level, with only Ghana and Liberia demonstrating significantly increased IPTp coverage with eight compared to four ANC contacts, and Nigeria demonstrating a negative correlation between ANC8 + and IPTp3 uptake.

Study findings may have potential implications for implementation of the WHO 2016 ANC policy guidelines. While the vast majority of pregnant women receive at least one ANC contact during their pregnancy, less than half of pregnant women receive at least four ANC contacts, and even fewer receive eight- the proportion of women attending eight ANC contacts exceeded 20% in only three countries, despite the fact that nearly a decade has elapsed since the policy recommendation. These findings highlight the need for countries to delve into the barriers to achieving higher ANC uptake in their country.

The WHO ANC policy recommendation included several WHO-recommended approaches to improve ANC attendance and outcomes in specific contexts [10]. First, the WHO recommends midwife-led continuity-of-care models in which a known midwife or small group of known midwives support a woman throughout the antenatal, intrapartum, and postnatal continuum. Deployment, training, and ongoing support of midwives, as well as monitoring for an adequate number of midwives needed to provide quality care, are essential in ensuring the success of this recommendation [10]. Second, the WHO advocates for packages of interventions that include household and community mobilization and antenatal home visits to improve ANC utilization, particularly in rural settings with low access to health services. To actualize this recommendation, robust health systems are needed to ensure continuity of care between community-based interventions and services provided at health facilities. While home visits may be considered an ANC contact, they do not necessarily replace the services received during formal ANC visits with a clinician. As a result, interventions that strengthen health systems should be implemented alongside these community-based interventions. Thirdly, the WHO calls for policy-makers to consider educational, regulatory, financial, and personal and professional support interventions to recruit and retain qualified health workers in rural and remote areas [14]. Health provider interventions to improve retention in such areas may increase access to health care services including ANC [15]. By reviewing whether they have employed any of these recommended strategies, countries may be able to identify next steps to take to improve coverage of ANC. To achieve success, global and local stakeholders in malaria and reproductive health must move beyond siloed policies and interventions to continually collaborate across technical areas and sectors.

Study findings also offer additional programmatic implications for national malaria control and reproductive health programmes. In the study countries, the overwhelming majority of women have at least one contact or receive at least one IPTp dose, but most still fall short of the recommended three doses. Additional strategies are needed to improve retention in ANC and consistent provision of IPTp. Potential interventions to be explored should include complementary demand generation activities and supply side interventions tailored to national contexts and current implementation and coverage status. Demand generation activities should move beyond knowledge about ANC attendance and IPTp and focus on sustaining these behaviours and addressing social and behavioural barriers to attendance. Evidence-based activities may include mHealth education activities [16], community outreach [17], community health worker involvement [18], engagement of community or religious leaders [19], and mother to mother support groups. Examples of supply side interventions to address structural barriers include provision of free and accessible high quality of care [6] and supply chain management [20]. Alternative models, such as Group ANC—where small numbers of women with similar gestational ages receive scheduled ANC together [15, 21, 22] and community IPTp delivery by trained community health workers may be helpful in certain contexts [23, 24].

Other promising and innovative behaviour change interventions need to be explored. In Côte d’Ivoire, a pilot intervention demonstrated improved provider behaviours, client satisfaction and uptake of ANC following the implementation of a suite of behavioural economics interventions including [i] “My pregnancy follow-up form” listing four essential services that pregnant women should receive, serving as both a reminder form for the service provider and as a checklist for the pregnant woman; [ii] [1] "Good Mom's Honor Roll" awarded to pregnant women who followed the entire ANC process from the first trimester, and iii] the “pregnancy follow-up certificate” awarded by the pregnant woman to the health center for having provided her with quality care throughout her pregnancy[25]. A “Pregnancy School” education concept designed by the Ghana Health Service but yet to be formally evaluated helps and prepares pregnant women and their families during their pregnancy journey [26]. The three parts of the School, run by government health facilities, teach about [i] healthy eating, [ii] danger signs and [iii] labor and delivery, respectively [26].

This analysis identified several areas requiring additional research. While countries may have adopted the 2016 policy at the national level, local implementation may vary significantly. Thus, additional exploration of the national to sub-national operationalization of the WHO policy at the national and sub-national levels may help identify opportunities for improvement. Future studies should also explore the social, financial and structural factors associated with eight ANC contacts across settings, including community norms, gender dynamics, and spousal support to identify barriers to accessing multiple ANC contacts. Additional efforts should explore positive deviants to determine factors relating to success in implementing policy changes and ensuring adherence to them. In addition, further social and behavioural change implementation research is needed to design effective interventions that enable women to overcome barriers to attendance.

Finally, while this study focused solely on the benefit of eight ANC contacts on IPTp uptake, additional research on other maternal and child health outcomes should be explored, per the rationale for the 2016 policy.

Study limitations include the fact that cross-sectional studies do not permit causal inferences regarding the associations examined here. Only national level data are presented; further exploration of sub-national ANC or IPTp trends and policies were not conducted. Unmeasured confounding remains an issue as only variables available in the DHS/MIS were included in the analysis. As such, other factors related to the uptake of ANC services such as health insurance coverage [27] and quality of care [28] were not explored. Finally, the study did not account for temporal and contextual factors such as the degree to which countries might have adopted or operationalized the policy before 2020 and the effect of the COVID19 pandemic after 2020.

## Conclusion

This study identified poor uptake of the WHO 2016 ANC policy, with only three countries achieving at least 20% coverage of ANC8 + . While pooled estimates supported a slightly higher likelihood of receiving IPTp3 among women who attended eight versus four ANC contacts,, this relationship was not consistent at country level, highlighting that improving the uptake of IPTp3 will require more than simply ensuring ANC attendance. Documenting the implementation guidance detailing best practices from countries that are successfully implementing the policy might improve the operationalization and ensure sustainable improvements in ANC and IPTp coverage. More effective collaboration between stakeholders across technical areas and sectors to move beyond siloed malaria and reproductive health policies and interventions will help improve implementation. This paper is a call to action to deliver the vision of the WHO and the global malaria community of a malaria free world. Achieving this dream requires not only relevant policies to improve ANC and IPTp coverage, but ensuring that they are operationalized by clear actionable guidance and local ownership.

### Supplementary Information

Additional file 1: Table 1: Description of Study Countries and Data Sources. Table 2: Pooled and Country Level Correlates of IPTp3.

## Data Availability

The datasets are available from the DHS Program web site, http://www.dhsprogram.com.

## Abbreviations

ANC: Antenatal Care
ANC1 +: At least one ANC contact
ANC4 +: At least four ANC contacts
ANC8 +: At least eight ANC contacts
AOR: Adjusted Odds Ratio
CI: Confidence Interval
DHS: Demographic Health Surveys
FANC: Focused Antenatal Care
IPTp: Intermittent Preventive Treatment in Pregnancy
IPTp1 +: At least one dose of IPTp
IPTp3 +: At least three doses of IPTp
IPTp6 +: At least six doses of IPTp
ITN: Insecticide-treated Nets
MIP: Malaria in pregnancy
MIS: Malaria Indicator Surveys
N/A: Not Applicable
Ref: Reference
WHO: World Health Organization
