# Supplementary material for: WHO antenatal care policy and prevention of malaria in pregnancy in sub-Saharan Africa
Source: Malar J. 2024 Jul 23;23:218. doi: 10.1186/s12936-024-05037-3 (PMC11264419; doi:10.1186/s12936-024-05037-3)
Supplement: Supplementary file 1 — Additional file 1: Table 1: Description of Study Countries and Data Sources. Table 2: Pooled and Country Level Correlates of IPTp3. [file 12936_2024_5037_MOESM1_ESM.docx]

# Supplemental Tables

## Supplemental Table 1: Description of Study Countries and Data Sources

| **Country** | **Survey** | **Year** | **Households** | **Women** |
| --- | --- | --- | --- | --- |
| Burkina Faso | DHS | 2021 | 5207 | 5999 |
| Cameroon | MIS | 2022 | 4113 | 4760 |
| Côte d’Ivoire | DHS | 2021 | 4844 | 5425 |
| Gabon | DHS | 2019-2021 | 2640 | 2922 |
| The Gambia | DHS | 2019-2021 | 2729 | 3777 |
| Ghana | MIS | 2019 | 2107 | 2308 |
| Guinea | MIS | 2021 | 1812 | 2147 |
| Kenya | MIS | 2020 | 2009 | 2064 |
| Liberia | DHS | 2019-2020 | 2561 | 2743 |
| Madagascar | DHS | 2021 | 5564 | 5734 |
| Mali | MIS | 2021 | 3256 | 4934 |
| Mauritania | DHS | 2019-2021 | 4355 | 5065 |
| Mozambique | MIS | 2018 | 3124 | 3377 |
| Niger | MIS | 2021 | 2394 | 2734 |
| Nigeria | MIS | 2021 | 4983 | 5497 |
| Senegal | DHS | 2020-2021 | 2188 | 3084 |
| Sierra Leone | DHS | 2019 | 4519 | 4899 |
| Tanzania | DHS | 2022 | 4581 | 4872 |
| Uganda | MIS | 2018-2019 | 4504 | 4826 |
| Zambia | DHS | 2018 | 4824 | 5191 |

## Supplemental Table 2: Pooled and Country Level Correlates of IPTp3

| **Country** | **AOR^a^ (95% CI) of IPTp3** | | | | | | | | | | | | | | |
| --- | --- | --- | --- | --- | --- | --- | --- | --- | --- | --- | --- | --- | --- | --- | --- |
|  | **0-3 ANC contacts (Ref: 4-7)** | **8+ ANC contacts (Ref: 4-7)** | **Early first ANC^b^** | **25-34 Years old (Ref: 15-24)** | **≥35 Years old (Ref: 15-24)** | **Rural^b^** | **Christian^b^** | **Literate^b^** | **ITN use^b^** | **Female HHH^b^** | **≥2 children under 5 years in HH^b^** | **Poorer HH (Ref: poorest)** | **Middle HH (Ref: poorest)** | **Richer HH (Ref: poorest)** | **Richest HH (Ref: poorest)** |
| Total | 0.36*** (0.36 0.38) | 1.06 (1.00 1.12) | 1.12*** (1.10 1.17) | 1.02 (0.99 1.06) | 1.01 (0.97 1.06) | 0.98 (0.95 1.03) | N/A | 1.01 (0.98 1.03) | N/A | 0.89*** (0.88 0.94) | 0.95** (0.92 1.00) | 1.07*** (1.03 1.12) | 1.11*** (1.06 1.17) | 1.11*** (1.06 1.17) | 1.18*** (1.11 1.25) |
| Burkina Faso | 0.42*** (0.37 0.47) | 1.15 (0.66 2.00) | 1.07 (0.96 1.21) | 1.20** (1.06 1.35) | 1.31*** (1.13 1.52) | 1.14 (0.97 1.34) | 1.13* (1.00 1.27) | 1.02 (0.90 1.17) | 1.12 (1.00 1.28) | 0.99 (0.82 1.20) | 0.93 (0.83 1.05) | 1.27** (1.08 1.50) | 1.34*** (1.13 1.57) | 1.48*** (1.24 1.77) | 1.76*** (1.40 2.22) |
| Cameroon | 0.51*** (0.44 0.60) | 1.02 (0.80 1.31) | 1.47*** (1.27 1.69) | 1.01 (0.88 1.17) | 1.10 (0.92 1.33) | 0.81* (0.68 0.97) | 0.94 (0.81 1.10) | 1.03 (0.87 1.23) | 1.31*** (1.14 1.50) | 1.06 (0.91 1.25) | 0.99 (0.86 1.14) | 1.05 (0.84 1.31) | 1.09 (0.86 1.39) | 1.22 (0.93 1.61) | 1.55** (1.15 2.10) |
| Côte d’Ivoire | 0.38*** (0.33 0.43) | 0.74 (0.54 1.03) | 1.08 (0.95 1.24) | 1.21** (1.05 1.39) | 1.30** (1.10 1.54) | 1.09 (0.94 1.28) | 1.13 (1.00 1.29) | 1.21** (1.05 1.40) | N/A | 1.01 (0.86 1.20) | 0.85** (0.75 0.96) | 1.05 (0.89 1.25) | 0.94 (0.78 1.14) | 1.19 (0.95 1.49) | 1.28 (0.99 1.68) |
| Gabon | 0.51*** (0.42 0.62) | 1.05 (0.80 1.40) | 1.09 (0.92 1.30) | 1.13 (0.95 1.35) | 1.03 (0.83 1.29) | 0.93 (0.77 1.13) | 1.13 (0.91 1.42) | 1.16 (0.92 1.46) | 1.24** (1.06 1.45) | 1.02 (0.87 1.21) | 1.09 (0.93 1.28) | 1.06 (0.85 1.33) | 1.30* (1.01 1.66) | 1.00 (0.76 1.33) | 1.18 (0.87 1.61) |
| The Gambia | 0.34*** (0.28 0.41) | 1.15 (0.84 1.57) | 0.91 (0.80 1.06) | 1.04 (0.90 1.22) | 0.83* (0.68 1.00) | 0.99 (0.82 1.21) | N/A | 1.07 (0.92 1.27) | 1.04 (0.92 1.20) | 1.14 (0.94 1.39) | 0.98 (0.82 1.17) | 1.05 (0.87 1.28) | 1.13 (0.91 1.42) | 1.31* (1.01 1.71) | 1.15 (0.85 1.56) |
| Ghana | 0.26*** (0.18 0.36) | 1.67*** (1.38 2.04) | 1.28* (1.05 1.57) | 1.50*** (1.20 1.87) | 1.45** (1.13 1.86) | 1.02 (0.81 1.28) | 1.50** (1.13 2.00) | 1.36** (1.11 1.66) | 1.05 (0.87 1.28) | 0.82 (0.68 1.01) | 1.07 (0.90 1.29) | 0.82 (0.64 1.08) | 0.77 (0.58 1.03) | 0.71* (0.51 0.99) | 0.70 (0.48 1.03) |
| Guinea | 0.29*** (0.24 0.35) | 1.01 (0.69 1.48) | 1.41*** (1.16 1.72) | 1.15 (0.95 1.42) | 1.10 (0.85 1.44) | 0.93 (0.67 1.31) | 0.60 (0.31 1.20) | 1.03 (0.81 1.33) | N/A | 1.15 (0.88 1.51) | 1.11 (0.91 1.37) | 1.32* (1.00 1.74) | 1.06 (0.81 1.42) | 1.08 (0.77 1.53) | 0.99 (0.64 1.54) |
| Kenya | 0.47*** (0.38 0.59) | 0.98 (0.58 1.65) | 1.42** (1.14 1.75) | 1.09 (0.87 1.37) | 1.24 (0.93 1.67) | 1.07 (0.85 1.35) | 0.93 (0.69 1.26) | 1.77*** (1.30 2.41) | 1.63*** (1.32 2.02) | 1.11 (0.90 1.38) | 1.39** (1.08 1.79) | 1.13 (0.85 1.51) | 0.86 (0.63 1.18) | 0.92 (0.68 1.26) | 0.69 (0.47 1.01) |
| Liberia | 0.43*** (0.33 0.56) | 1.43*** (1.18 1.72) | 1.03 (0.86 1.25) | 0.93 (0.78 1.12) | 1.07 (0.87 1.33) | 1.12 (0.91 1.38) | 1.17 (0.95 1.46) | 0.90 (0.76 1.08) | 1.13 (0.97 1.33) | 1.01 (0.86 1.21) | 1.21* (1.04 1.42) | 1.17 (0.96 1.44) | 1.11 (0.88 1.42) | 0.97 (0.71 1.33) | 0.85 (0.60 1.21) |
| Madagascar | 0.41*** (0.36 0.47) | 0.73 (0.48 1.12) | 1.14 (1.00 1.30) | 0.99 (0.88 1.13) | 1.10 (0.93 1.30) | 1.14 (0.96 1.38) | 0.84** (0.74 0.96) | 1.36*** (1.18 1.57) | 1.57*** (1.38 1.80) | 1.07 (0.93 1.25) | 0.90 (0.80 1.02) | 1.05 (0.89 1.25) | 1.31** (1.09 1.57) | 1.13 (0.94 1.39) | 0.80 (0.63 1.03) |
| Mali | 0.26*** (0.23 0.31) | 1.06 (0.69 1.66) | 1.43*** (1.24 1.65) | 1.07 (0.93 1.24) | 0.99 (0.83 1.19) | 1.19 (0.94 1.51) | 0.99 (0.68 1.45) | 1.20* (1.01 1.42) | 0.96 (0.83 1.13) | 1.06 (0.79 1.43) | 1.06 (0.88 1.28) | 1.41** (1.14 1.75) | 1.71*** (1.39 2.11) | 1.58*** (1.27 1.97) | 1.51** (1.11 2.06) |
| Mauritania | 0.52*** (0.43 0.64) | 1.08 (0.71 1.68) | 0.93 (0.77 1.14) | 1.12 (0.89 1.41) | 1.27 (0.99 1.64) | 1.04 (0.82 1.34) | N/A | 0.80* (0.65 0.98) | 1.41** (1.14 1.73) | 0.96 (0.79 1.18) | 1.12 (0.90 1.42) | 0.95 (0.71 1.27) | 1.23 (0.93 1.64) | 0.76 (0.52 1.10) | 0.88 (0.58 1.35) |
| Mozambique | 0.28*** (0.24 0.33) | 0.87 (0.56 1.37) | 1.01 (0.85 1.23) | 0.86 (0.73 1.01) | 0.76** (0.62 0.93) | 0.95 (0.79 1.16) | N/A | 1.11 (0.93 1.33) | 1.31** (1.08 1.58) | 1.08 (0.92 1.27) | 1.15 (1.00 1.33) | 1.00 (0.79 1.28) | 0.83 (0.65 1.07) | 1.03 (0.81 1.34) | 1.10 (0.82 1.49) |
| Niger | 0.31*** (0.25 0.38) | 4.99 (0.99 25.25) | 1.30* (1.04 1.61) | 1.22 (0.99 1.51) | 1.27 (0.97 1.66) | 1.74** (1.16 2.59) | N/A | 1.01 (0.77 1.34) | 1.12 (0.84 1.53) | 1.15 (0.81 1.65) | 1.16 (0.94 1.44) | 1.88*** (1.37 2.57) | 1.87*** (1.37 2.55) | 1.82*** (1.33 2.50) | 2.42*** (1.55 3.76) |
| Nigeria | 0.30*** (0.26 0.34) | 0.68*** (0.56 0.81) | 2.09*** (1.83 2.39) | 0.87 (0.76 1.02) | 1.02 (0.86 1.23) | 0.81** (0.70 0.94) | 0.98 (0.85 1.14) | 1.11 (0.95 1.31) | 1.31*** (1.15 1.49) | 0.99 (0.79 1.25) | 1.01 (0.89 1.16) | 1.14 (0.92 1.42) | 1.58*** (1.27 1.96) | 1.48*** (1.17 1.87) | 1.69*** (1.30 2.19) |
| Senegal | 0.46*** (0.36 0.57) | 0.31 (0.04 2.53) | 1.32* (1.05 1.66) | 1.03 (0.83 1.28) | 1.13 (0.88 1.47) | 0.76* (0.60 0.97) | 1.21 (0.64 2.28) | 1.41*** (1.15 1.73) | 0.73** (0.60 0.89) | 0.80* (0.64 1.00) | 1.05 (0.83 1.34) | 0.97 (0.76 1.25) | 0.73* (0.54 0.97) | 0.64* (0.46 0.91) | 0.66* (0.45 0.97) |
| Sierra Leone | 0.56*** (0.48 0.66) | 0.93 (0.81 1.09) | 1.01 (0.90 1.15) | 0.95 (0.83 1.09) | 0.92 (0.78 1.09) | 0.87 (0.71 1.09) | 0.71 (0.04 11.73) | 1.01 (0.87 1.17) | 1.22** (1.07 1.40) | 0.72*** (0.62 0.83) | 0.93 (0.83 1.05) | 1.27** (1.07 1.50) | 1.44*** (1.21 1.73) | 0.97 (0.76 1.26) | 0.80 (0.60 1.09) |
| Tanzania | 0.63*** (0.54 0.74) | 1.35 (0.94 1.96) | 1.44*** (1.23 1.67) | 1.15 (1.00 1.35) | 0.94 (0.78 1.14) | 0.66*** (0.55 0.79) | N/A | 1.35*** (1.14 1.61) | 1.25** (1.07 1.47) | 1.07 (0.92 1.26) | 0.79*** (0.69 0.90) | 1.02 (0.83 1.27) | 0.89 (0.73 1.12) | 0.63*** (0.50 0.80) | 0.76* (0.58 0.98) |
| Uganda | 0.56*** (0.49 0.63) | 1.00 (0.63 1.60) | 1.08 (0.96 1.23) | 0.85* (0.74 0.97) | 0.81* (0.68 0.95) | 0.98 (0.85 1.14) | N/A | 0.92 (0.81 1.06) | 1.13 (0.99 1.30) | 0.92 (0.81 1.07) | 1.09 (0.97 1.23) | 1.05 (0.89 1.24) | 1.02 (0.85 1.23) | 1.11 (0.91 1.36) | 1.19 (0.96 1.48) |
| Zambia | 0.42*** (0.37 0.48) | 1.45 (0.78 2.69) | 1.08 (0.96 1.24) | 1.11 (0.98 1.27) | 1.04 (0.89 1.23) | 0.80* (0.67 0.96) | 0.99 (0.61 1.63) | 1.21** (1.07 1.37) | 1.29*** (1.15 1.45) | 0.89 (0.78 1.04) | 1.18** (1.05 1.33) | 1.11 (0.95 1.31) | 1.30** (1.09 1.55) | 1.22 (0.98 1.54) | 1.60*** (1.23 2.08) |
| Abbreviations: AOR: Adjusted Odds Ratio; CI: Confidence Interval; HH: Household; HHH: Head of Household; N/A: Not Available; Ref: reference. ^a^Adjusted for early first ANC, age group, rural residence, religion, reading literacy, insecticide treated net use, sex of head of household, number of children under five in household and wealth quintile.  ^b^Reference (Ref) group is the null (no category) | | | | | | | | | | | | | | | |
